# Social isolation in Covid-19: The impact of loneliness

**DOI:** 10.1177/0020764020922269

**Published:** 2020-04-29

**Authors:** Debanjan Banerjee, Mayank Rai

**Affiliations:** 1 Department of Psychiatry, National Institute of Mental Health and Neurosciences (NIMHANS), Bengaluru, India; 2 Department of Psychiatry, Regional Institute of Medical Sciences (RIMS), Imphal, India

‘All of humanity’s problems stem from the man’s inability to sit quietly in a room alone’.

We need to revisit this statement by Blaise Pascal time and again to unearth something invaluable, to reinforce something primal, especially in times such as these where the whole world is in a state of lockdown, courtesy the corona virus disease 2019 (COVID-19). This disease caused by SARS-CoV-2, has literally brought the world down to its knees just within last few months.

## COVID-19

The world is facing a global public health crisis for the last three months, as the coronavirus disease 2019 (COVID-19) emerges as a menacing pandemic. Besides the rising number of cases and fatalities with this pandemic, there has also been significant socio-economic, political and psycho-social impact. Billions of people are quarantined in their own homes as nations have locked down to implement social distancing as a measure to contain the spread of infection. Those affected and suspicious cases are isolated. This social isolation leads to chronic loneliness and boredom, which if long enough can have detrimental effects on physical and mental well-being. The timelines of the growing pandemic being uncertain, the isolation is compounded by mass panic and anxiety. Crisis often affects the human mind in crucial ways, enhancing threat arousal and snowballing the anxiety. Rational and logical decisions are replaced by biased and faulty decisions based on mere ‘faith and belief’. This important social threat of a pandemic is largely neglected. We look at the impact of COVID-19 on loneliness across different social strata, its implications in the modern digitalized age and outline a way forward with possible solutions to the same.

There is no doubt that national and global economies are suffering, the health systems are under severe pressure, mass hysteria has acquired a frantic pace and people’s hope and aspirations are taking a merciless beating. The uncertainty of a new and relatively unknown infection increases the anxiety, which gets compounded by isolation in lockdown. As global public health agencies like World Health Organization (WHO) and Centre for Disease Control and Prevention (CDC) struggle to contain the outbreak, social distancing is repeatedly suggested as one of the most useful preventive strategies. It has been used successfully in the past to slow or prevent community transmission during pandemics (WHO, 2019). While certain countries like China have just started recovering from their three-month lockdown, countries like Iran, Italy and South Korea have been badly hit irrespective of these measures and those like India have initiated nation-wide shutdown and curfews to prevent the community transmission of COVID-19. Ironically however, the social distancing is a misnomer, which implies physical separation to prevent the viral spread.

The modern world has rarely been so isolated and restricted. Multiple restrictions have been imposed on public movement to contain the spread of the virus. People are forced to stay at home and are burdened with the heft of quarantine. Individuals are waking up every day wrapped in a freezing cauldron of social isolation, sheer boredom and a penetrating feeling of loneliness. The modern man has known little like this, in an age of rapid travel and communication. Though during the earlier outbreaks of Severe Acute Respiratory Syndrome (SARS), Middle East Respiratory Syndrome (MERS), Spanish flu, Ebola and Plague the world was equally shaken with millions of casualties, the dominance of technology was not as much as to make the distancing felt amplified (Smith, 2006). In this era of digitalization, social media, social hangouts, eateries, pubs, bars, malls, movie theatres to keep us distracted creating apparent ‘social ties’. Humankind has always known what to do next, with their lives generally following a regular trail. But this sudden cataclysmic turn of events have brought them face to face with a dire reckoning – how to live with oneself. It is indeed a frightening realization when a whole generation or two knows how to deal with a nuclear fallout but are at their wit’s end on how to spend time with oneself. Ironically, however, it has stranded them with their families (those who are unaffected by the illness) and are expected to strengthen the bonds of relationship. But, as mentioned before, the ‘virtual connectedness’ provided by social media has probably made us forget what proximity in relationships feel like. This can be a double-edged sword, that can either mend or strain relations, based on the pre-existing intimacy and communication patterns. It feels like a monumental task to stay stuck with yourself and your loved ones, while the pandemic looms large over the world.

## Loneliness during a pandemic: the impact and social variations

Loneliness is often described as the state of being without any company or in isolation from the community or society. It is considered to be a dark and miserable feeling, a risk factor for many mental disorders like depression, anxiety, adjustment disorder, chronic stress, insomnia or even late-life dementia (Wilson et al., 2007). Loneliness is common in the old-age group, leading to increased depression rates and suicide. It has been well-documented that long periods of isolation in custodial care or quarantine for illness has detrimental effects on mental well-being (Stickley & Koyanagi, 2016). Loneliness is proposed to break this essential construct and disrupt social integration, leading to increase in isolation. This is a vicious cycle which makes the lonely individual more segregated into his own ‘constricted’ space. Loneliness is also one of the prime indicators of social well-being (Cacioppo & Patrick, 2008). Most people cringe at the idea of this social isolation. They will do anything to keep themselves preoccupied or distracted, from acts of outrageous indulgences to preposterous shows of vanity and depravation. Besides, loneliness has also shown to be an independent risk factor for sensory loss, connective tissue and auto-immune disorders, cardio-vascular disorders and obesity. If this self-isolation and lockdown is prolonged, it is likely that chronic loneliness will decrease physical activity leading to increased risk of frailty and fractures (Mushtaq et al., 2014).

This COVID-19 pandemic seems to have brought our frenzied speed of modern society to a grinding halt and has literally crushed the wings of unlimited social interaction. Under these social restrictions, individuals are forced to reconcile with this terrifying reality of isolation which can contribute to domestic inter-personal violence and boredom. Similar trends of increase in isolation and loneliness have been noticed among emergency workers and quarantined population in Wuhan, China. This has increased the prevalence of depression, anxiety, post-traumatic stress disorders and insomnia in the population. It also contributes to fatigue and decreases performance in health-care workers (Torales et al., 2020). But neither life nor the society had probably readied us for this task. The concept of boredom and loneliness leads to anger, frustration on the authorities and can lead for many to defy the quarantine restrictions, which can cause dire public health consequences. Emotional unpreparedness for such biological disasters have detrimental effects, as this situation is unprecedented in all measures. It also makes us take a step back and question: is social distancing only for a specific social class; as millions of migrant labourers, homeless individuals and daily wage workers stay stranded in their workplaces, railway and bus stations and factories with overcrowding and poor hygiene. When basic amenities of life are scarce, it is far-fetched myth to think about distancing or hand sanitization according to the prescribed standards (The Print, 2020; www.theprint.in). Isolation or loneliness for them is thus different. It is being away from their origins, their families and being deprived of basic human rights and self-dignity. Segregation from self-identity can also form the basis for loneliness, just that it reflects differently in different socio-economic strata (Valkenburg & Peter, 2008). It is again ironic, how the construct of loneliness varies based on the social strata giving rise to dimensional psycho-social needs.

## The way forward

First step in this journey is to transform this devious loneliness to solitude. Loneliness, which on one hand is an emotion filled with terror and desolation, solitude, its cousin is full of peace and tranquillity. The primal answer to loneliness has always been in our roots: the ability to be at peace with oneself. This however has been a habit long lost by the humanity in the trends of globalization.

Many great works of art, philosophy, literature have emerged from solitude. This comes with enjoying one’s existence and ability to cherish the bonds with others. This might be a good time to engage in long-forgotten hobbies, neglected passions and unfulfilled dreams. Improving proximal bonds with family and loved ones is another opportunity. Distancing from social media will be beneficial, as during times of pandemic it can contribute to ‘infodemic’ causing information overload. COVID-19 by all means is a ‘digital epidemic’ where the related statistics spread faster than the virus itself. Only relevant and updated information about the situation outside helps relieve anxiety during isolation (Hyvärinen & Vos, 2016). It is vital that the virus does not invade us ‘psychologically’ which can last much beyond the resolution of this pandemic.

As mental health professionals, we need to be sensitive to the personalized needs of those in quarantine and cater to them. Their personal and psychological needs are to be adhered to. Digital communication needs to be maintained with their loved ones. As mentioned, before social connectedness matters. Similar protocols in China during the first stage of outbreak had shown to improve quality of lives of those isolated (Duan & Zhu, 2020). Need for community-based and brief psycho-social interventions have also been stressed upon by Torales et al. (2020) in their recent article, acknowledging the chronic mental health impact of the ongoing pandemic situation. Furthermore, research has shown that as simple as weekly telephonic sessions can help reduce anxiety at the time of pandemics. These sessions need to be brief and solution-focused (Yang et al., 2020). Social integration forms another important aspect, in which involvement of the associated people in life matters. Taking care of the domestic helpers, the vendors, the security personnel, etc. or even a simple exchange of greetings with neighbors or strangers can give a feeling that ‘we are all in this together’. The bonds of humanity turn even more important at such times, when the whole world shares the same threads of anxiety. Similar sensitization needs to be done for the allied specialities to understand and appreciate the mental health needs of a biological disaster. The pandemic will eventually be over giving rise to two important lessons: the emotional preparedness for solitude at times of such crisis and psycho-social well-being forming the cornerstone of public health.
